# Hydroxyurea-induced membrane fluidity decreasing as a characterization of neuronal membrane aging in Alzheimer’s disease

**DOI:** 10.18632/aging.202949

**Published:** 2021-05-11

**Authors:** Qiujian Yu, Xiaoqin Cheng

**Affiliations:** 1 Department of Neurology, First Affiliated Hospital of Kunming Medical University, Kunming 650000, China; 2 Department of Neurology, Zhongshan Hospital, Fudan University, Shanghai 200032, China

**Keywords:** Alzheimer's disease, hydroxyurea, membrane aging, β-amyloid, DHA

## Abstract

Aging is one of the significant risk factors for Alzheimer’s disease (AD). Therefore, this study aimed to propose a new hypothesis “membrane aging” as a critical pathogenesis of AD. The concept of “membrane aging” was reviewed, and the possible mechanisms of membrane aging as the primary culprit of AD were clarified. To further prove this hypothesis, a hydroxyurea-induced “membrane aging” model was established *in vitro* and *in vivo*. First, neuronal aging was validated by immunocytochemistry with age-related markers, and membrane aging phenotypes were confirmed. The alterations of membrane fluidity within APP/PS1 mice were re-proved by intracerebroventricular injection of hydroxyurea. Decreased membrane fluidity was found *in vitro* and *in vivo*, accompanied by increased total cholesterol concentration in neurons but decreased cholesterol levels within membrane fractions. The Aβ level increased considerably after hydroxyurea treatment both *in vitro* and *in vivo*. DHA co-treatment ameliorated membrane aging phenotypes and Aβ aggregation. The study revealed the AMP-activated protein kinase/acetyl CoA carboxylase/carnitine palmitoyl transferase 1 pathway involved in membrane aging processes. These results strongly supported the idea that membrane aging was a pathogenesis of AD and might serve as a new therapeutic target for AD.

## INTRODUCTION

Alzheimer’s disease (AD) is the most common cause of dementia. Its manifestations include cognitive impairment and dysfunction of language and perceptual skills, learning and memory ability, orientation and problem-solving functions, and so forth. Approximately 90% of patients are diagnosed with late-onset AD in people aged more than 65 years; the remaining 10% are younger individuals diagnosed with early-onset AD [1]. Neurodegenerative diseases, especially AD, are now of great concern, posing heavy economic burden for individuals, families, and even the whole society. In Mainland China, around 6.3 people in every 1000 are diagnosed with AD every year [2].

Aging is the most noteworthy risk factor for AD. Multiple components such as genetic instability, DNA damage, and free radical accumulation and waste lead to and/or accelerate aging [3–6]. In senescence, the membrane aging process consists of age-associated physiological changes in the membrane such as abnormal activities of enzymes and receptors, alterations of membrane potential, disturbance of membrane lipid composition, and, in particular, changes in membrane fluidity [7, 8]. Reduced membrane fluidity was presumed to be a manifestation of membrane aging. It inhibited normal membrane functions such as cytoskeleton anchoring, cellular transportation, intracellular communication, or neural transmission. Additionally, glucose metabolism was disrupted, finally resulting in the aggregation of Aβ. In conclusion, a change in neuronal membrane fluidity could be critical pathogenesis of AD.

To prove this hypothesis, a hydroxyurea-induced aging model was established in this study. Hydroxyurea (HU) is a medication used in sickle-cell disease. It is used as an antineoplastic drug; it suppresses the ribonucleotide reductase and inhibits the generation of deoxyribonucleotides [9]. Therefore, it can trigger DNA double-strand breaks (DSBs) near the replication sites and mitochondrial DNA, leading to cellular and mitochondrial stress and dysfunction and further provoking senescence-like alterations in diverse cell lines [10, 11]. In the present study, decreased membrane fluidity was found *in vitro* and *in vivo*, accompanied by increased Aβ aggregation. The total cholesterol concentration in neurons increased, and the cholesterol levels in membrane fractions decreased. After administering docosahexaenoic acid (DHA), the membrane fluidity increased and Aβ aggregation was partly reversed. Furthermore, the AMP-activated protein kinase (AMPK)/acetyl CoA carboxylase (ACC)/carnitine palmitoyl transferase 1 (CPT1) pathway was found to be involved in the process of membrane aging.

## MATERIALS AND METHODS

### Primary neuronal cell cultures

Rat primary cortical neurons were cultured as described previously [12]. Briefly, sufficient coverslips coated with poly-D-lysine (Millipore, USA) were fully prepared before culture. The brains were removed form postnatal day 1 (P1) Sprague–Dawley (SD) rat pups, and cortexes were gently dissected. Then, 0.25% trypsin (Gibco, USA) was added to the dissection medium and incubated with the cortical tissue in a water bath for 20 min; then, 0.5 mL of DNase (Sigma, USA) solution was applied for 5 min. Tissues were now loosened by trypsin incubation. The tissue was washed twice and suspended with the Neurobasal medium supplemented with B27 and GlutaMAX (Invitrogen, USA). The dissociated primary neurons were plated into prepared coverslips. The plating medium was refreshed after incubating at 37° C for 4 h. Cytoarabine (araC) (Selleck, China) was added to a final concentration of 2μM 2 days after plating to inhibit non-neuronal differentiation and maintained for 48 h. The medium was replaced with maintenance medium (Neurobasal medium supplemented with B27); half of the medium was replaced twice a week with fresh maintenance medium warmed to 37° C.

### Hydroxyurea treatment

The cells were seeded at a density of 1 × 10^6^ cells per mL in a 35-mm dish (Corning Incorporated, NY, USA). Hydroxyurea (HU) (Sigma, MO, USA) was applied at a concentration of 0.5, 4, 8, and 16mM and incubated at 37° C until the collection time points.

### Cell viability detection

The cells were seeded at approximately 5 × 10^3^ cells per mL in 96-well plates (Corning Incorporated, NY, USA). Cell viability was detected using a Cell Counting Kit-8 (CK04-11, Dojindo Laboratories, Japan) after treatment with different concentrations of HU within various durations following the manufacturer’s protocol.

### Immunocytochemistry

The cells were fixed with 4% paraformaldehyde for 15 min. After several washes, they were blocked with 0.01M phosphate-buffered saline supplemented with 1% BSA and 0.3% Triton X-100 for 30 min. Primary antibodies (mouse anti-LAP2α, 1:500, Abcam, UK; mouse anti-betaIII tubulin, 1:500, Abcam; mouse anti-NeuN, 1:1000, Abcam; rabbit anti-NeuN, 1:200, Abways; rabbit anti-Tbr1, 1:500, Abcam; mouse anti-γH2AX, 1:250, Millipore, USA; mouse anti-Lamin A/C, 1:200, Abcam; rabbit anti-H3K9me3, 1:4000, Abcam; mouse anti-6E10, 1:200, Abcam) were diluted with blocking buffer and applied overnight at 4° C. The cells were stained with suitable Alexa Fluor–labeled secondary antibodies (Invitrogen) in blocking buffer at a concentration of 1:500 for 1.5 h at room temperature. 4', 6-Diamidino-2-phenylindole (Thermo Fisher, IL, USA) was applied to counterstain cells for visualizing nuclei at 1:1000 in Milli Q water (Biocel, Millipore, USA). The images were obtained with an Olympus IX71 microscope using a Hamamatsu ORCA CCD camera and Leica TCS SP5.

### Neuronal cholesterol detection

The cells were seeded in 96-well plates (Corning Incorporated, NY, USA). Total cholesterol was visualized following the instruction on the cholesterol detection kit (cell-based) (Bio Vision, CA, USA). The images were obtained with an Olympus IX71 microscope using a Hamamatsu ORCA CCD camera.

### Preparation of native cortical and hippocampal membranes

Rat cortical and hippocampal tissues were separated and homogenized in buffer A (4mM HEPES + 0.32M sucrose + protein inhibitor). The homogenate was centrifuged at 1000*g* for 10 min at 4° C. The resultant supernatant was then centrifuged at 10,000*g* for 20 min at 4° C. This procedure was repeated until the supernatant was clear. The final pellet (native membranes) was suspended in a minimum volume of buffer B (50mM Tris, pH 7.4). The protein concentration was assayed using a BCA assay kit as the standard.

### Membrane fluidity determinations

Membrane fluidity was determined with three fluorescent probes: 1, 6-diphenyl-1,3,5-hexatriene (DPH), N,N,N-trimethyl-4-(6-phenyl-1,3,5-hexatrien-1-yl) phenylammonium *p*-toluenesulfonate (TMA-DPH) and 1,3-di-2-pyrenyl propane (DPP).

Briefly, the cells were seeded at a density of approximately 5 × 10^3^ cells per mL in 96-well plates (Corning Incorporated). DPH and TMA-DPH (AAT Bioquest, CA, USA) were applied at the concentrations of 2μM and 5μM in PBS and tetradrofuran anhydrous (Sigma), respectively, following the manufacturer’s protocol. The membrane fluidity was detected using SoftMax Pro 5.4.1 following the protocol of basic FP-anisotropy with the excitation polarization light at 355 nm, and emission was collected at 430 nm.

The following formula was used:

*r* = (*I*_V_ – *GI*_H_)/(*I*_V_ + 2*GI*_H_)

where *I*_V_ and *I*_H_ are fluorescence intensities determined at vertical and horizontal orientations of the emission polarizer when the excitation polarizer is set in the vertical position.

*G* is the correction factor. *R* values were calculated and were inversely proportional to membrane variability.

DPP fluorescent probe was diluted in ethanol of spectroscopic grade and mixed with the cell medium at a final concentration of 12.5nM. The cells were incubated in the dark at 4° C for 5 h to achieve maximal incorporation of the fluorescent probe to the membranes. The fluorescence of DPP in membranes was detected at 24° C on a Perkin Elmer fluorescence spectrometer, LS50B. The fluorophore was excited at 329 nm, and the monomer and excimer fluorescence intensities were read at 379 and 480 nm, respectively. The excimer to monomer fluorescence intensity ratio (*I*e/*I*m) was then calculated, which was positively related to membrane fluidity.

### Determinations of the cholesterol levels in the neuronal membrane

The membrane pellets were acquired from a 60-mm dish (Corning Incorporated). The neurons were lysed in MES buffer (25mM, pH 7.1), and the membrane pellets were obtained via ultracentrifugation of the extract at 70,000 rpm (TLA 100.1 rotor, Beckman) at 4° C for 1 h. The cholesterol levels were determined using an Amplex Red cholesterol assay kit (Invitrogen-A12216, USA). The fluorescence levels in the final AmplexRed membrane pellet solution were detected using a Perkin Elmer fluorescence spectrometer, LS50B.

### Western blot analysis

Rat primary cortical neurons were cultured for 5 days and lysed in radioimmunoprecipitation assay buffer (Sigma–Aldrich, USA) with complete protease inhibitor mixture (Roche, Switzerland). Equal amounts of each lysate were fractionated by 10% SDS-PAGE and electroblotted onto 2-μm nitrocellulose-based transfer membrane. The membranes were blocked with 5% blocking solution (50 mL of TBST with 2.5 g Marvel dried skimmed milk powder) for 1 h, followed by incubation with primary antibodies [rabbit anti-AMPKα1, 1:1000, CST, USA; rabbit anti-AMPKα2, 1:1000, CST; rabbit anti-ACC1, 1:1000, CST; rabbit anti-phospho ACC (Ser79), 1:1000, CST; rabbit anti-phospho AMPKα1 (Ser485)/AMPKα2 (Ser491), 1:1000, CST; mouse anti-CPT1c, 1:500, Abcam; mouse anti-β actin, 1:1000, Abcam] at 4° C overnight. The membranes were washed with TBST (1× TBS and 0.1% Tween 20) three times and incubated with secondary antibodies for 1 h at room temperature. Followed by several washes, the membranes were analyzed, and images were captured using an Odyssey infrared fluorescence imaging system (LI-COR, USA).

### RNA isolation and quantitative reverse transcription–polymerase chain reaction

The cells were lysed with TRIzol (Life Technologies, USA), treated for DNA contamination, and reverse-transcribed using a QuantiTect RT kit (Qiagen, Germany). The mRNA levels were assayed using an SYBR Green PCR kit (Qiagen) on a MasterCycler RealPlex2 (Eppendorf, Germany). All results were normalized to a β-actin control. The primers used are listed in Table 1.

**Table 1:** PCR primers.

**Genes of interest**	**Gene ID**	**Sequence (5’→3’)**
SLC19A2	289175	Forward CACCGAAATCGCCTACTACTC Reverse GAGCCCACTGTAAAGCCCA
SLC19A3	316559	Forward ACTCTTGGGTTTATCCCACTGT Reverse CGTTGCCAGGTAAGAATATGTCC
SLC22A2	29503	Forward CAGCTTGTCTATACAGCCTTGC Reverse CTGTGTAGTTGGCAGGATGTT
SLC22A3	29504	Forward GTGGATGTCAGAGTCGGCTC Reverse CCGCTTTCGTAAGATGGGTGT
AMPKα1	65248	Forward GTCAAAGCCGACCCAATGATA Reverse CGTACACGCAAATAATAGGGGTT
AMPKα2	78975	Forward TCGCAGTGGCTTATCATCTC Reverse TGTCGTATGGTTTGCTCTGG
ACC1	60581	Forward TGAGGAGGACCGCATTTATC Reverse GCATGGAATGGCAGTAAGGT
CPT1c	308579	Forward TCTTCACTGAGTTCCGATGGG Reverse ACGCCAGAGATGCCTTTTCC
β- actin	11461	Forward GGCTGTATTCCCCTCCATCG Reverse CCAGTTGGTAACAATGCCATGT
GAPDH	14433	Forward AGGTCGGTGTGAACGGATTTG Reverse TGTAGACCATGTAGTTGAGGTCA

### Animals and general housing conditions

Male SD rats (both 18 months old and 2 months old), APP/PS1 transgenic mice (6 months old), and wild-type control mice (6 months old) (obtained from Shanghai Model Organisms Center, Inc.) were used in the present study.

Male rats used in this study were divided into two groups: aging (18 months old) and young group (2 months old); six rats in each group were studied. A total of 12 APP/PS1 transgenic mice and 12 wild-type control mice were used (3 mice per group). All experimental procedures involving animals were performed according to the guidelines established by the ethics committee for the use of experimental animals in Shanghai Fudan University, and every attempt was made to limit animal numbers and suffering. All animals were housed at 20–25° C and had free access to food and water during a 12-h light/dark cycle.

### Intracerebroventricular injection in mice

APP/PS1 transgenic mice and wild-type control mice were injected with 3 μL of HU (32μM) or 0.9% normal saline (NS) bilaterally into the lateral ventricles with the injection rate of 0.3 μL/min twice a week for 4 weeks, using a 10-μL Hamilton syringe (Hamilton, Reno, NV) with a 31-gauge needle (Hamilton) as previously described [13]. In brief, the mice were anesthetized with 2% pentobarbital sodium until their activity slowed. The needle was inserted perpendicularly into the skull surface 0.6 mm backward and 1.6 mm leftward/rightward of the bregma, with a depth of 2.6 mm. After bilateral injection of the ventricles, the mice were rewarmed slowly by placing them on a warming pad until they were warm and active. The injected mice were then returned to the normal housing condition.

### Immunohistochemical analyses *in vivo*

APP/PS1 transgenic and wild-type control mice were perfused with 4% paraformaldehyde. The brains were removed, postfixed overnight at 4° C, cryoprotected with 30% sucrose in phosphate-buffered saline for 48 h, mounted in OCT embedding compound, and frozen. Coronal sections (10 μm in thickness) were cut using a cryostat. Immunohistochemistry was performed by incubation with the anti-β-amyloid antibody (1:1000, Previously Covance, USA). The sections were washed, incubated for 2 h with a biotinylated secondary antibody (1:200, Vector Laboratories, CA, USA), and visualized using a Peroxidase Substrate DAB Kit (Vector Laboratories).

### Image analyses

GraphPad Prism 6.0 software (GraphPad Software, Inc., USA) and ImageJ 7.0 software were used for the analyses and quantification of immunohistochemical signals, respectively.

### Statistical analyses

All statistical analyses were performed using GraphPad Prism 6.0 software (GraphPad Software, Inc., USA). The results were expressed as mean ± standard error of the mean. Differences between groups were assessed with the Student *t* test for two groups and one-way analysis of variance (ANOVA) for multiple group comparisons. *P* values were calculated, and a *P* value <0.05 indicated a significant difference.

## RESULTS

### Establishment and characterization of cell senescence in rat primary cortical neurons

**Figure 1:**
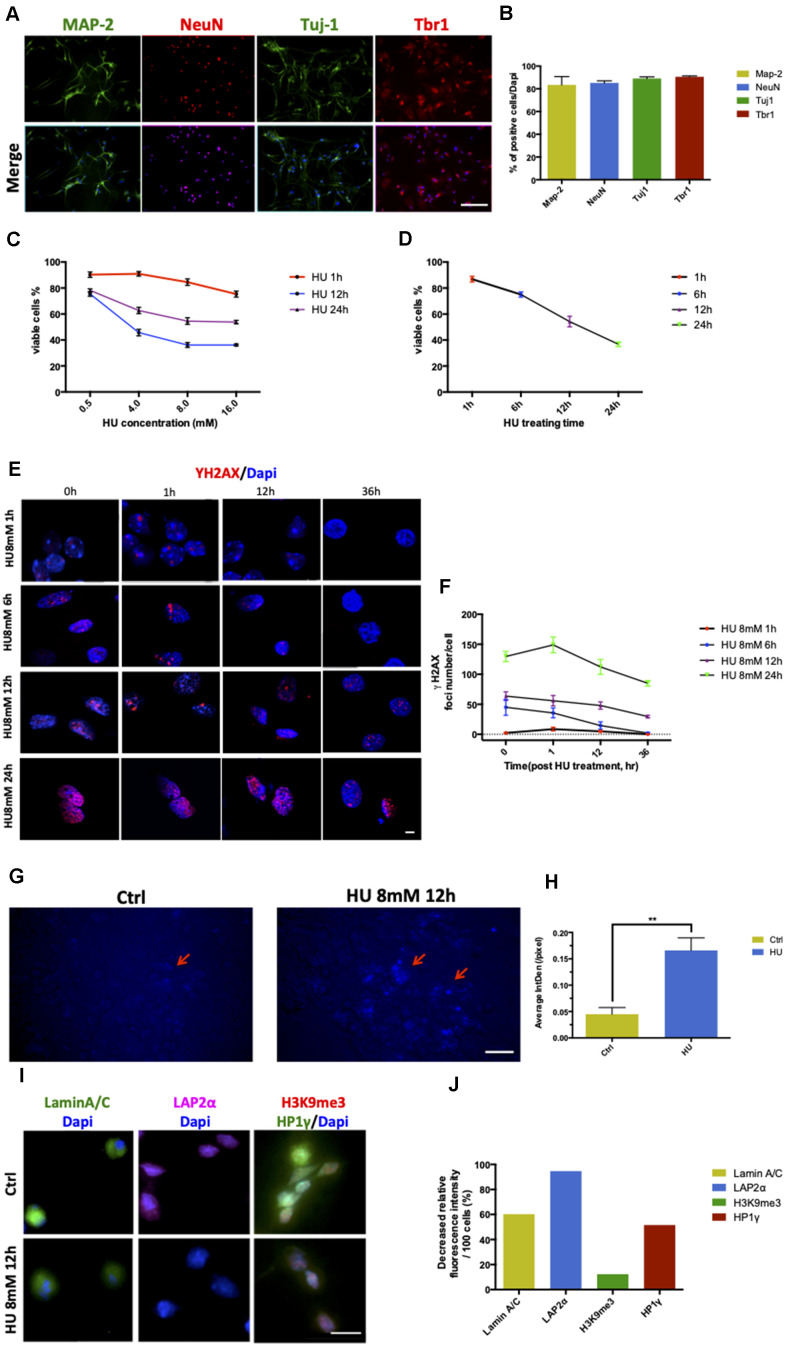
**Establishment and characterization of neuronal senescence.** More than 80% of rat primary cortical neurons (cultured till day 5) were positively stained with neuronal markers of MAP-2 (green), NeuN (red), Tju1 (green), and Tbr1 (red). Scale bar: 200 μm (**A**, **B**). Dose-dependent and time-dependent curves represented neuronal viability using CCK8 test detection (**C**, **D**). Formation of DNA double-strand breaks in rat primary cortical neurons (5 days) after HU treatment. Neurons were treated with 8mM HU for 1, 6, 12, and 24 h and allowed to recover until 36 h after treatment. The cells were fixed and stained for γH2AX foci (red) at the indicated time points after HU treatment. Scale bar: 5 μm (**E**). Graphical quantification of the number of γH2AX foci in neurons treated with 8mM HU for different times over 36 h (**F**). *In vitro* staining of total cholesterol (blue) in the 12-h 8mM HU treatment group compared with the control neurons. Scale bar: 500 μm (**G**). Average fluorescent intensity of the cholesterol-positive cells among control and HU-treated cells. As shown earlier, HU treatment significantly increased the level of blue-staining intensity (*P* < 0.01) (**H**). Immunocytochemistry for markers identifying the nuclear lamina (Lamin A/C), a lamina-associated protein (LAP2α), and peripheral heterochromatin (3K9me3, HP1γ) in HU-treated neurons compared with the control ones. Scale bar: 25 μm (**I**). Quantification of the markers depicted in (**I**) demonstrated the decreased relative fluorescence intensity per 100 cells in the HU group compared with the control group (**J**). All the data are expressed as mean ± SD from three independent experiments (*N* = 3). ^*^P < 0.05, ^**^P < 0.01, ^***^P < 0.001. The Student *t* test was used to determine the statistical significance of the differences.

The cortical regions of the brain were removed from postnatal day 1 (P1) SD rats and cultured in the presence of B27 and Neurobasal medium *in vitro*. After 5 days, the neurons presented their mature characteristics and more than 80% stained positive for Map-2, NeuN, Tuj1, and Tbr1 (Figure 1A, 1B).

The neurons were treated with HU for 1, 6, 12, and 24 h at four different doses of 0.5, 4, 8, and 16mM, respectively, to determine the optimal time/dose of HU treatment so as to induce cell senescence (Figure 1C, 1D). The dose- and time-dependent curves of the CCK8 test showed that the percentage of viable cells was more than 60% after treatment with 8mM HU for 12 h, which was acceptable to further study cellular aging caused by HU treatment.

Positive γH2AX staining foci represent the sites of DNA damage. By fixing the HU concentration at 8mM, the neurons treated for 1 and 6 h showed an increase in the number of γH2AX foci, which declined rapidly and returned to almost no damage 36 h after treatment. The treatment with 8mM HU for 12 and 24 h led to a fast increase in the number of γH2AX foci, which was maintained even 36 h after treatment. However, the former time point of treatment was more optimal for neurons *in vitro*, causing relatively mild damages compared with the latter (Figure 1E, 1F).

The total cholesterol concentrations in neurons were detected by cholesterol staining. HU-treated neurons showed more positive staining, with significantly increased fluorescence intensity compared with the control (*P* < 0.01) (Figure 1G, 1H).

In addition, HU-treated neurons showed abnormalities in nuclear morphology, such as loss of the nuclear structural organization marker (Lamin A/C), loss of the nuclear lamina-associated protein LAP2α, and global loss of the heterochromatin markers tri-methylated H3K9 (H3K9me3) and heterochromatin protein 1 gamma (HP1γ) (Figure 1I, 1J). The decreased age-related markers supported the phenotypes of cellular aging.

### Establishment and validation of “membrane aging” *in vivo* and *in vitro*

**Figure 2:**
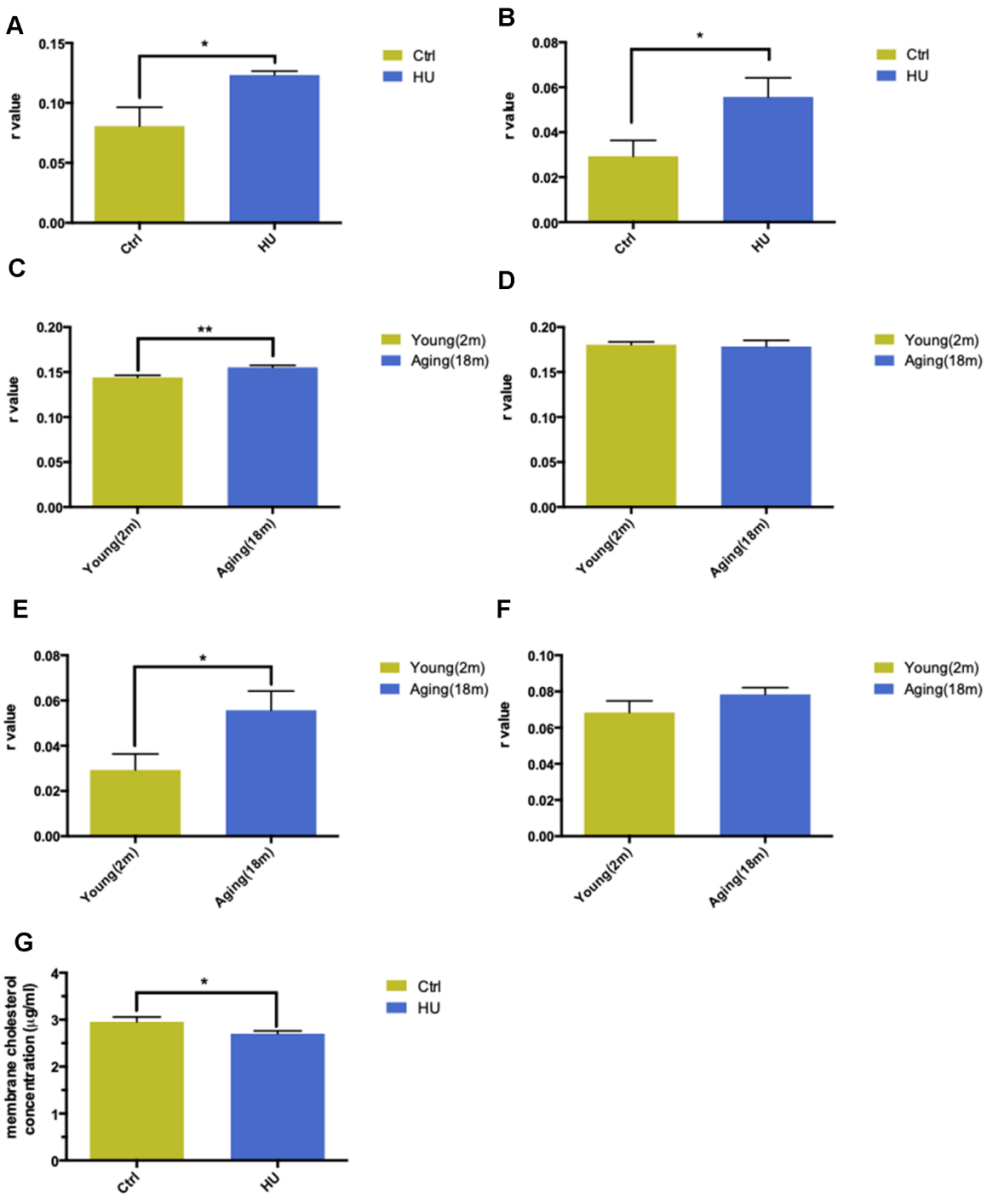
**Validation of “membrane aging” *in vivo* and *in vitro*.** Changes in membrane fluidity and cholesterol levels in membrane pellets are two main characteristics of membrane aging. Fluorescent probe polarization is one of the most direct measurements representing the alterations in membrane fluidity. HU treatment significantly increased the *r* value compared with the control *in vitro* via applying the DPH fluorescent probe (*P* < 0.05) (**A**). The same trend was observed while probing with TMA-DPH (*P* < 0.01) (**B**). The results demonstrated that HU treatment decreased neuronal membrane fluidity dramatically because the *r* value was inversely proportional to membrane mobility. *In vivo*, native membrane pellets were carefully extracted, followed by hippocampus and cortex isolation in 18-month-old male SD rats (*n* = 6) and 2-month-old male SD rats (*n* = 6). When probing with DPH in cortical membrane pellets, the *r* value increased significantly in the aging group compared with the young group (*P* < 0.01) (**C**). In hippocampal regions, no difference was observed between two groups (**D**). Similar results were obtained by applying a TMA-DPH fluorescent probe in cortical and hippocampal native membrane pellets *in vivo*. The *r* value considerably increased in elderly rats only in the cortical regions (*P* < 0.05) but without apparent changes in the hippocampus (**E**, **F**). Regarding the alterations in membrane lipid composition, HU-treated neurons showed a decreased cholesterol level in neuronal membrane pellets (*P* < 0.05) (**G**). All the data are expressed as mean ± SD from three independent experiments (*N* = 3). ^*^P < 0.05, ^**^P < 0.01, ^***^P < 0.001. The Student *t* test was used to determine the statistical significance of the differences.

With regard to the aging process, the change in biomembrane fluidity may be a major mechanism of age-related structural changes and brain function deterioration. The membrane fluidity was determined using DPH and TMA-DPH as fluorescent probes. The former tends to be a “classical” fluorescent hydrophobic probe, whereas the latter is a trimethylamino-modified derivative of DPH, with a very rapid incorporation time in plasma membranes. HU-treated aging neurons showed a significantly higher r value compared with the control group when applying both probes, which represented lower membrane fluidity (P < 0.05). In addition, TMA-DPH seemed more sensitive in distinguishing membrane variability between the two groups (Figure 2A, 2B).

The hippocampal and cortical regions from aging (18 months old, n = 6) and young SD male rats (2 months old, n = 6) were dissected to further validate the results *in vivo*. After native membrane isolation, two probes were used again to detect membrane fluidity in the two regions. Surprisingly, the study revealed that aging rats showed dramatically decreased membrane fluidity only in the cortical membranes (*P* < 0.001) instead of in the hippocampus via administering the DPH fluorescent probe (Figure 2C, 2D). Same results were obtained via applying TMA-DPH (*P* < 0.05) (Figure 2E, 2F).

The cholesterol level in neuronal membrane pellets was assessed to study the changes in membrane lipid composition. HU-treated neurons showed significantly lower levels of cholesterol compared with the control ones (*P* < 0.05) (Figure 2G).

### Validation of membrane aging in APP/PS1 transgenic mice

**Figure 3:**
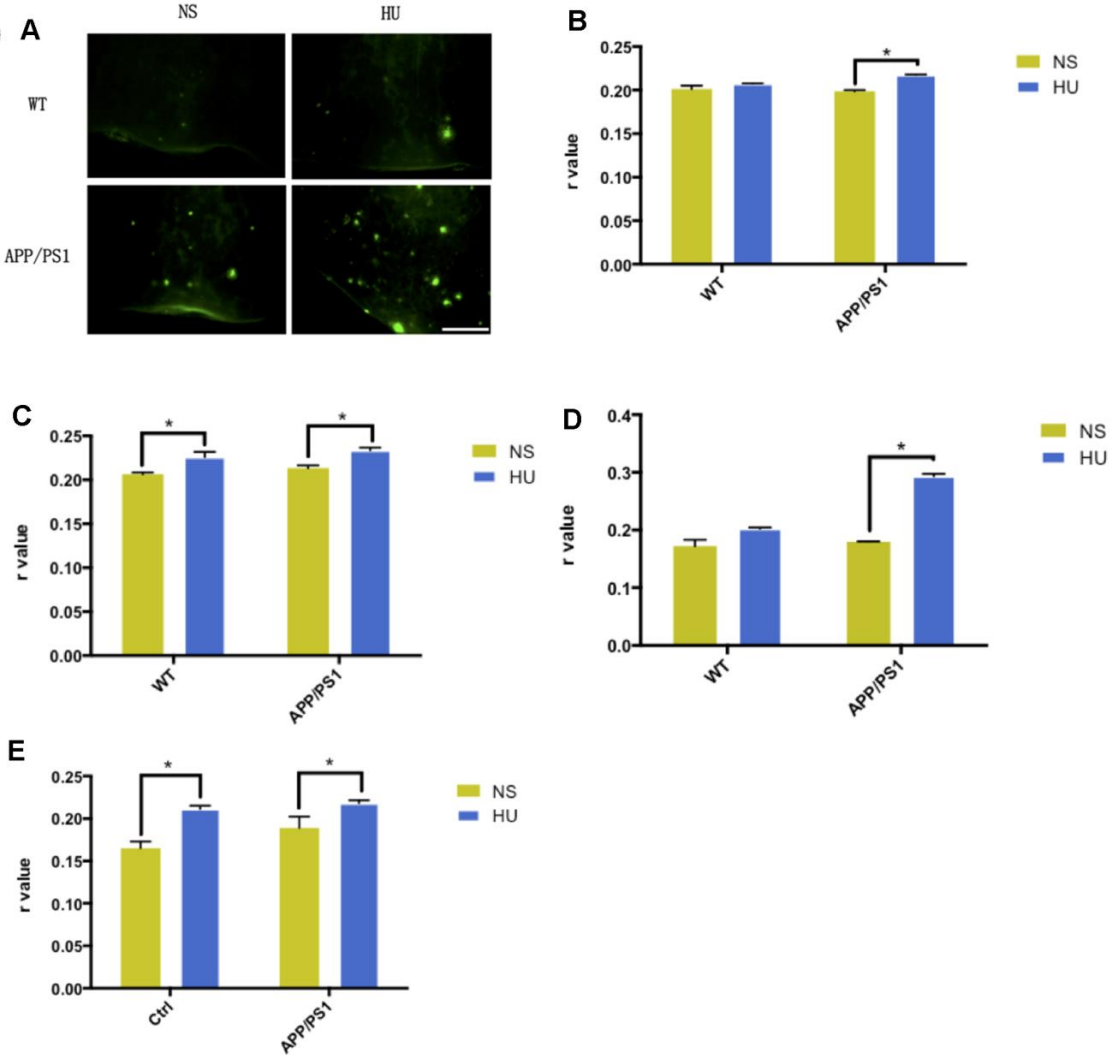
**Validation of membrane aging in APP/PS1-mutated transgenic mice.** In the dissected cortical region, APP/PS1-mutated transgenic mice showed accelerated Aβ deposition after HU intracerebroventricular injection compared with mice with NS injection and wild-type controls. Scale bar: 1 mm (**A**). For the membrane fluidity determination probed with TMA-DPH and DPH, 32μM HU injection dramatically increased *r* values in the hippocampal and cortical membranes of APP/PS1 mice compared with NS-injected ones (*n* = 3, *P* < 0.05). Same trends were found only in hippocampal membranes in wild-type controls (*n* = 3, *P* < 0.05) (**B**–**E**). All the data are expressed as mean ± SD from three independent experiments (*N* = 3). ^*^P < 0.05. Two-way ANOVA was used to determine the statistical significance of the differences.

Increased Aβ aggregation was found after bilateral ventricular injection of HU in APP/PS1 mutated transgenic mice compared with mice injected with NS and wild-type controls, in both cortical and hippocampal regions (Figure 3A, 3B).

Significant higher *r* values were found for membrane fluidity determination probed with TMA-DPH and DPH after HU injection in the hippocampal and cortical membranes of APP/PS1 mice compared with NS-injected ones (*n* = 3, *P* < 0.05). It was established that intracerebroventricular HU injection decreased membrane fluidity in the mouse brain. Same results were found only in hippocampal membranes in wild-type controls (*n* = 3, *P* < 0.05) (Figure 3C–3F).

### Membrane aging phenotypes was ameliorated by DHA intervention

Various concentrations of DHA were applied to aging neurons in the membrane for 12 h to confirm the effects of DHA on membrane fluidity. The results were detected using two fluorescent probes: TMA-DPH and DPP. TMA-DPH was used for its higher sensitivity instead of DPH, whereas DPP was introduced because it anchored predominantly between the acyl chains of fatty acids in the membrane hydrocarbon core, which could be distinguished from the former one.

**Figure 4:**
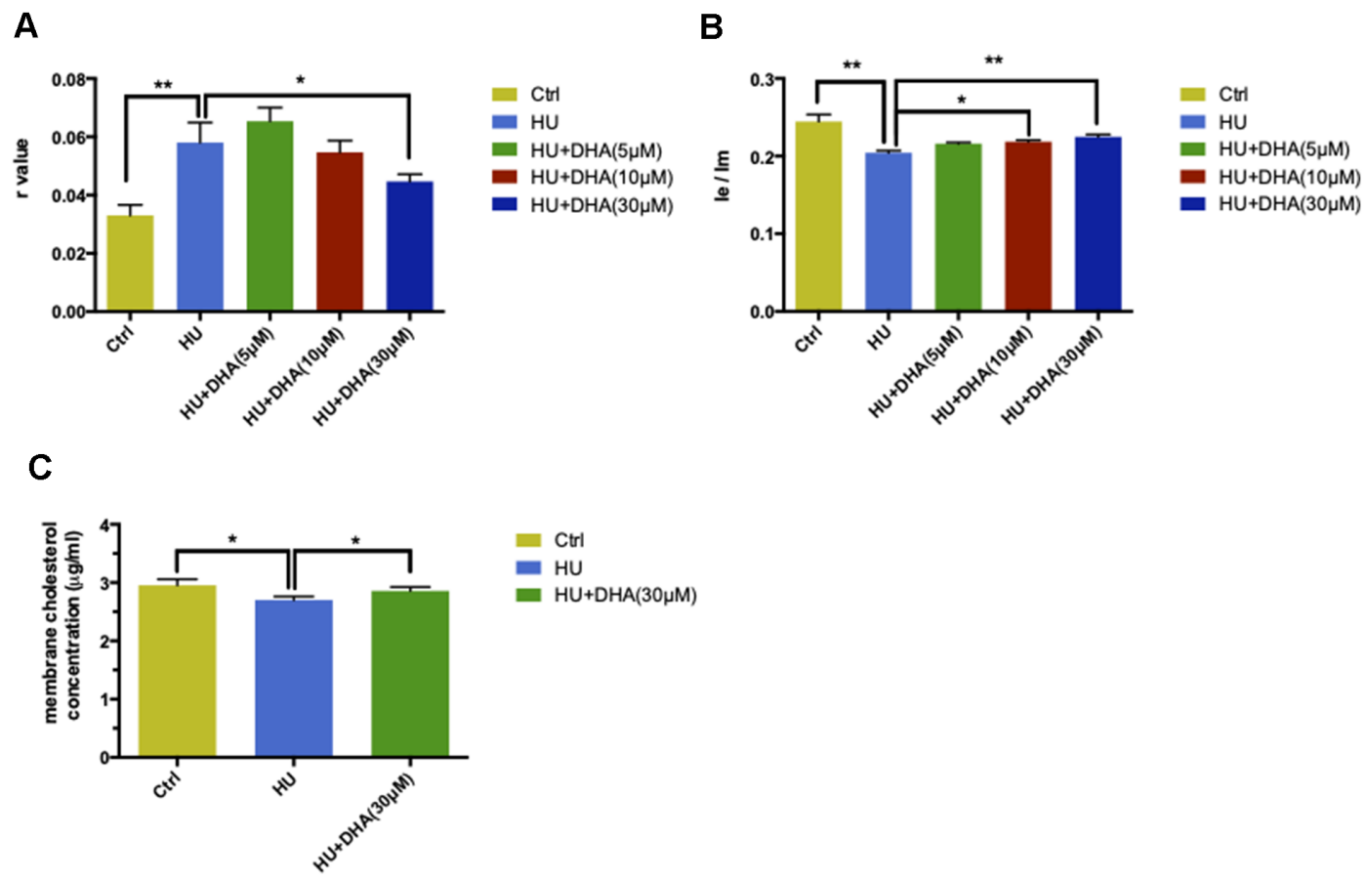
**DHA intervention ameliorated membrane aging phenotypes.** TMA-DPH and DPP were used as two fluorescent probes to detect membrane mobility *in vitro*. In the TMA-DPH probing experiment, HU treatment considerably increased the *r* value compared with the control (*P* < 0.01). However, co-treatment with different concentrations of DHA gradually decreased the *r* value. Especially after 30μM DHA treatment, the *r* value showed a dramatic decline compared with HU treatment (*P* < 0.05) (**A**). Similar results were confirmed by the DPP probing test. HU treatment decreased the *I*e/*I*m ratio noticeably (*P* < 0.01). The ratio gradually increased after co-treatment with the increasing concentrations of DHA. *I*e/*I*m was considerably augmented after treatment with 10μM and 30μM DHA compared with HU treatment (*P* < 0.05 and *P* < 0.01) (**B**). The *r* value is inversely proportional to membrane fluidity, while the ratio of *I*e/I*m* is directly proportional to membrane mobility. Regarding membrane lipid composition *in vitro*, the cholesterol level in membrane pellets significantly increased in the 30μM DHA co-treatment group compared with HU treatment group (**C**). All the data are expressed as mean ± SD from three independent experiments (*N* = 3). ^*^P < 0.05, ^**^P < 0.01. Student *t* test and one-way ANOVA were used to determine the statistical significance of the differences.

In the TMA-DPH probing experiment, HU treatment increased the *r* value compared with the control (*P* < 0.05). This was followed by co-treatment with DHA at concentrations ranging from 5μM to 30μM; a significant decrease in the *r* value was observed in the 30μM DHA co-treatment group compared with the HU group (*P* < 0.05) (Figure 4A). In the DPP probing test, the result of *I*e/*I*m was in direct proportion to membrane fluidity. HU treatment dramatically decreased the ratio of *I*e/*I*m (*P* < 0.01). Then, the ratio gradually increased after treatment with increased concentrations of DHA; the changes in *I*e/*I*m were observed after co-treatment with 10μM and 30μM DHA compared with single HU administration (*P* < 0.05 and *P* < 0.01) (Figure 4B). Taken together, DHA administration improved membrane fluidity in aging neurons. Particularly, 30μM DHA was more effective.

The cholesterol level in membrane pellets in primary cortical neurons was quantified to explore whether the lipid composition could also be altered by DHA application. After co-treatment with 30μM DHA, the cholesterol levels in the membrane increased significantly compared with HU treatment alone (*P* < 0.05) (Figure 4C).

In conclusion, DHA intervention could ease membrane aging phenotypes by increasing membrane fluidity and escalating cholesterol concentration.

### AD phenotypes appeared in the neuronal membrane aging model

Two classical characterizations exist for AD: extracellular deposition of Aβ and intracellular aggregation of neurofibrillary tangles.

**Figure 5:**
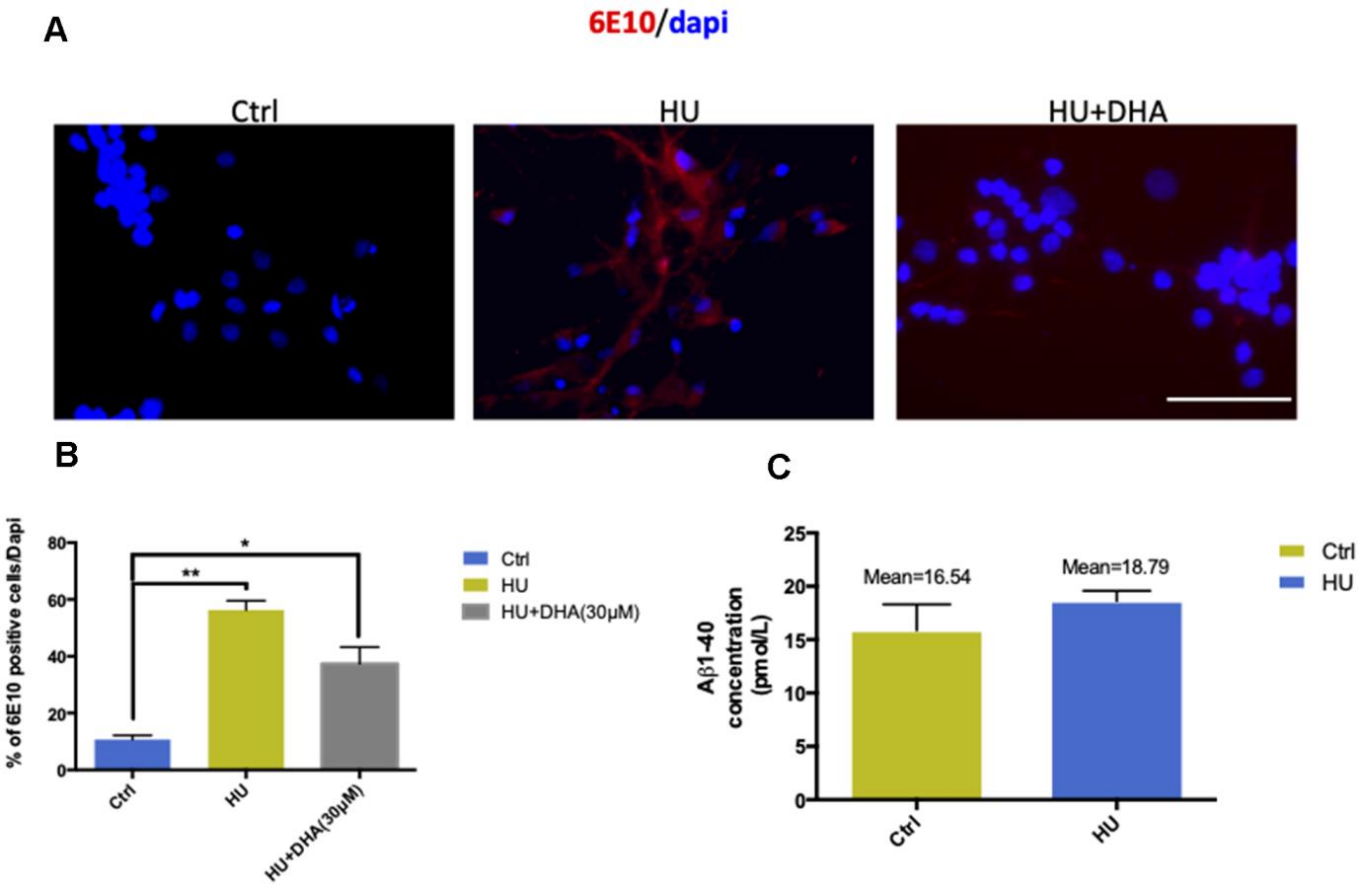
**Membrane aging increased Aβ aggregation *in vitro*.** The results of immunostaining with 6E10 showed that membrane aging significantly increased the number of positively stained neurons compared with the control (*P* < 0.01). After co-treatment with DNA, Aβ aggregation decreased (**A**, **B**). Further quantification of Aβ *in vitro* was done by ELISA detection. The average concentration of Aβ1-40 was at least 2 pmol/L higher in the membrane aging group compared with the control group (**C**). However, Aβ1-42 was under the detection threshold, which was hard to quantify. All the data are expressed as mean ± SD from three independent experiments (*N* = 3). ^*^P < 0.05, ^**^P < 0.01. The Student *t* test was used to determine the statistical significance of the differences.

In membrane aging neurons, 6E10 was used to detect Aβ depositions. The results showed that Aβ staining was positive in the membrane aging model compared with the control (Figure 5A, 5B). ELISA was used for further quantification. The mean concentration of Aβ1-40 was approximately 2 pmol/L higher in the HU group compared with the control group (Figure 5C). However, Aβ1-42 was hardly detected in both groups, implying that the concentrations of Aβ1-42 were under the detection threshold.

Also, the mRNA levels of Aβ1-40 and Aβ1-42 were detected *in vivo*. The results showed that Aβ42 levels increased in the cortex and hippocampus of HU-treated APP/PS1 mice, and the mRNA levels of Aβ1-40 and Aβ1-42 considerably increased (Supplementary Figure 1A–1D).

### Underlying mechanisms of the AMPK/ACC/CPT1 pathway in membrane aging processes

**Figure 6:**
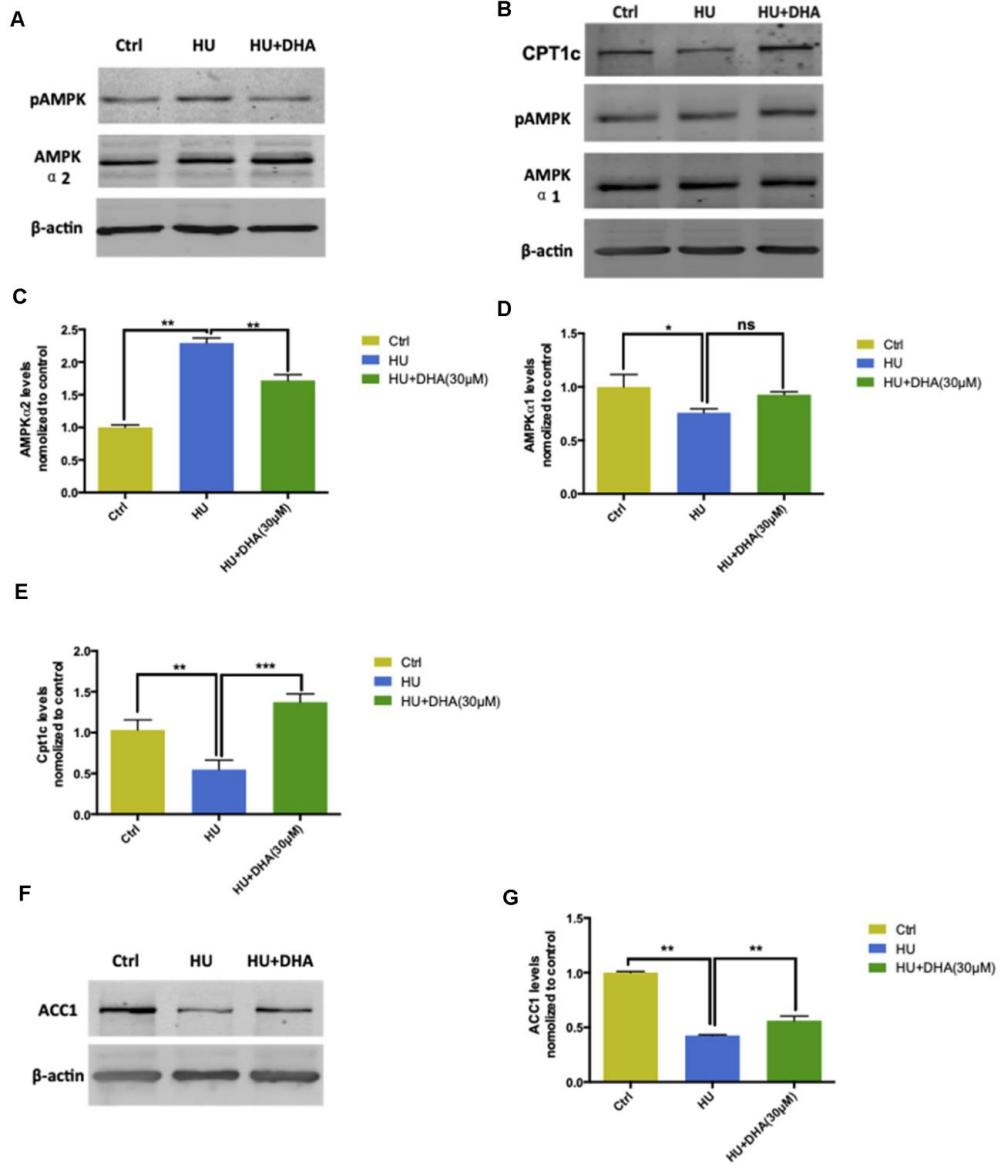
**AMPK/ACC/CPT1 pathway was involved in membrane aging processes.** Membrane aging activated protein expression of AMPK-α2 through Ser485/491 residue phosphorylation by DHA treatment. However, AMPK-α1 showed no significant change in the protein expression level (**A**, **B**). Membrane aging also increased AMPK-α2 mRNA levels (*P* < 0.01), and DHA co-treatment partly decreased mRNA levels of AMPK-α2 (*P* < 0.01) (**C**). AMPK-α1 mRNA levels decreased in the membrane aging group and slightly increased in the DHA treatment group (**D**). CPT1c protein and mRNA levels were also inhibited by membrane aging (*P* < 0.01) and eased by DHA intervention (*P* < 0.01) (**B**, **E**). The protein and mRNA expression of two downstream factors of AMPK were detected. Membrane aging inhibited the expression of ACC1 protein; DHA co-treatment comparatively increased the protein level (**F**). The ACC1 mRNA level followed the same trend, which was reduced by membrane aging (*P* < 0.05) and then ameliorated by DHA administration (*P* < 0.01) (**G**). All the data are expressed as mean ± SD from three independent experiments (*N* = 3). ^*^P < 0.05, ^**^P < 0.01, ^***^P < 0.001. One-way ANOVA was used to determine the statistical significance of the differences.

AMPK expression and ACC1 and CPT1c levels were detected using Western blot analysis and q-PCR. Membrane aging activated AMPK-α2 via phosphorylating Ser485/491 residues and was further dephosphorylated by DHA intervention (Figure 6A). In contrast, HU treatment had no effect on AMPK-α1; however, DHA administration increased the phosphorylation of AMPK-α1 (Figure 6B). The aforementioned results were further verified by detecting the mRNA levels of AMPK-α1 and AMPK-α2. Membrane aging increased the mRNA levels of AMPK-α2 considerably (*P* < 0.01); after co-treatment with DHA, the AMPK-α2 level decreased dramatically (*P* < 0.01) (Figure 6C). The mRNA level of AMPK-α1 was reduced by membrane aging (*P* < 0.05) and increased by DHA administration (Figure 6D). ACC1 and CPT1c are two downstream factors of AMPK, which take part in lipid synthesis. The ACC1 level was detected by Western blot analysis and verified by q-PCR. Membrane aging reduced ACC1 expression *in vitro*, which was partly rescued by DHA interference (Figure 6E). The mRNA level of ACC1 showed a similar trend in the membrane aging group (*P* < 0.001), and was further increased by DHA co-treatment (*P* < 0.001) (Figure 6F). In addition, membrane aging reduced the expression of CPT1c, and was also rescued by DHA administration (Figure 6G); the result was further verified by mRNA expression (*P* < 0.001) (Figure 6H).

## DISCUSSION

Aging and aging-related diseases characterize a dramatic social and economic burden to society. Aging is one of the significant risk factors for AD progression. Previous studies proposed AD as a metabolic disorder. It was assumed that the dysfunction of brain glucose metabolism as well as Aβ deposition contributed to AD pathogenesis [14, 15]. However, glucose metabolism disorder could only be considered as a secondary factor. It was hypothesized that membrane aging could be critical pathogenesis of AD.

In terms of membrane aging, membrane fluidity was explored in this study. Membrane fluidity can be described as the viscosity of the lipid bilayer of the cell membrane, which is considered as a core-ascribing factor of membrane aging [16]. Multiple elements contribute to the aging process, such as DNA damage, disorder of nuclear organization, loss of heterochromatin, accumulation of free radicals, and so forth [3–6, 17, 18]. HU was used to provoke senescence-like alterations in neurons *in vitro* and *in vivo* [10, 11]. In the present study, rat primary cortical neurons were treated with 8mM HU for 12 h, and a stress-induced membrane aging model was successfully established. HU treatment not only induced membrane aging phenotypes, such as decreased membrane fluidity *in vitro* and *in vivo*, and altered membrane lipid composition, but also showed increased senescence-related DNA damage, total cholesterol level, and positive staining of age-related markers.

Lower membrane mobility of different aging samples might be an underlying biogenetic factor for neurodegenerative diseases such as AD, Huntingdon disease, and so forth [19]. A similar result was obtained by Scheuer and his colleagues. They collected AD frontal cortex and parietal cortex samples and found a slight reduction in the membrane fluidity of the parietal cortex. Such alteration specifically complied with the histopathological modifications of AD [20]. Van Rensvurg et al. indicated that membrane fluidity increased in platelets and decreased in erythrocytes in patients with AD [21].

On the contrary, cholesterol plays a crucial role in modulating the biophysical properties of cell membranes, especially membrane fluidity. Therefore, a change in the cholesterol level in membrane pellets is another contributor to membrane aging. For instance, Nakamura and his colleagues reported that membrane cholesterol levels decreased in senescent TIG-1 cells, accompanied by increased total cholesterol levels *in vitro* [22]. This study demonstrated increased total cholesterol levels in HU-treated neurons but decreased cholesterol levels in membrane fractions.

Docosahexaenoic acid (DHA) is an omega-3 fatty acid, which is a primary structural component of the human brain, cerebral cortex, skin, and retina. In humans, DHA is either obtained from the diet or may be converted in small amounts from eicosapentaenoic acid via docosapentaenoic acid as an intermediate [23]. Of note, as a precursor of endogenous proresolving lipid mediators, DHA may have a protective potential in controlling chronic inflammation and may further participate in maintaining normal membrane functions and reversing the decrease in membrane fluidity. Therefore, it may become a potential etiology-based therapy for AD [24]. An increasing body of evidence indicates a positive correlation between DHA concentrations and brain cognition [25]. Since 50% of the weight of a neuron's plasma membrane is composed of DHA, it may further participate in maintaining normal membrane functions [26]. This study demonstrated that DHA considerably eased the decreasing membrane fluidity in HU-treated groups. These results strongly supported the idea that DHA had neuroprotective functions, especially in membrane fluidity *in vitro* and *in vivo*. In addition, increased positive staining and mRNA levels of Aβ were found in membrane aging models *in vitro* and *in vivo* compared with the control.

Last but not least, this study revealed that the AMPK/ACC/CPT1 pathway participated in membrane aging processes. AMPK is a serine-threonine kinase known to be a major metabolic sensor that plays a role in maintaining metabolic homeostasis [27]. AMPK plays a central role in controlling lipid metabolism through modulating the downstream ACC and CPT1 pathway [28]. The AMPK/ACC/CPT1 pathway is found throughout the body, including brain regions [29]. This study revealed that membrane aging activated AMPKα2 but not AMPKα1 and inactivated its downstream target ACC1. In addition, the activity of CPT1c was inhibited by ACC1 inactivation. The changes in the activity of the AMPK/ACC/CPT1 pathway in the neuronal membrane aging model might disturb membrane lipid synthesis and provoke membrane lipid oxidation, further contributing to the changes in membrane fluidity.

In conclusion, this study established a membrane aging model *in vitro* and *in vivo*, which could largely duplicate the membrane aging phenotypes (e.g., decreased membrane fluidity and cholesterol concentration of membrane pellets). Based on this model, the neuroprotective function of DHA intervention was identified. Furthermore, the study showed that the AMPK/ACC/CPT1 pathway contributed to membrane aging processes, thus explaining the changes in membrane fluidity in the membrane aging model due to lipid synthesis disorders. The present study strongly supported the previous hypothesis that membrane aging was critical pathogenesis of AD.

### Availability of data and materials

All data and material are available.

### Consent for publication

All the authors listed have approved the manuscript that is enclosed.

### Ethics approval and consent to participate

All animal care and experimental procedures were approved by Medical Experimental Animal Administrative Committee of Fudan University and by Medical Experimental Animal Administrative Committee of First Affiliated Hospital of Kunming Medical University, China.

## Supplementary Material

Supplementary Figure 1
