# Supplementary material for: Hydroxyurea-induced membrane fluidity decreasing as a characterization of neuronal membrane aging in Alzheimer’s disease
Source: Aging (Albany NY). 2021 May 11;13(9):12817–32. doi: 10.18632/aging.202949 (PMC8148445; doi:10.18632/aging.202949)
Supplement: Supplementary Figure 1 [file aging-13-202949-s001.pdf]

SUPPLEMENTARY FIGURE

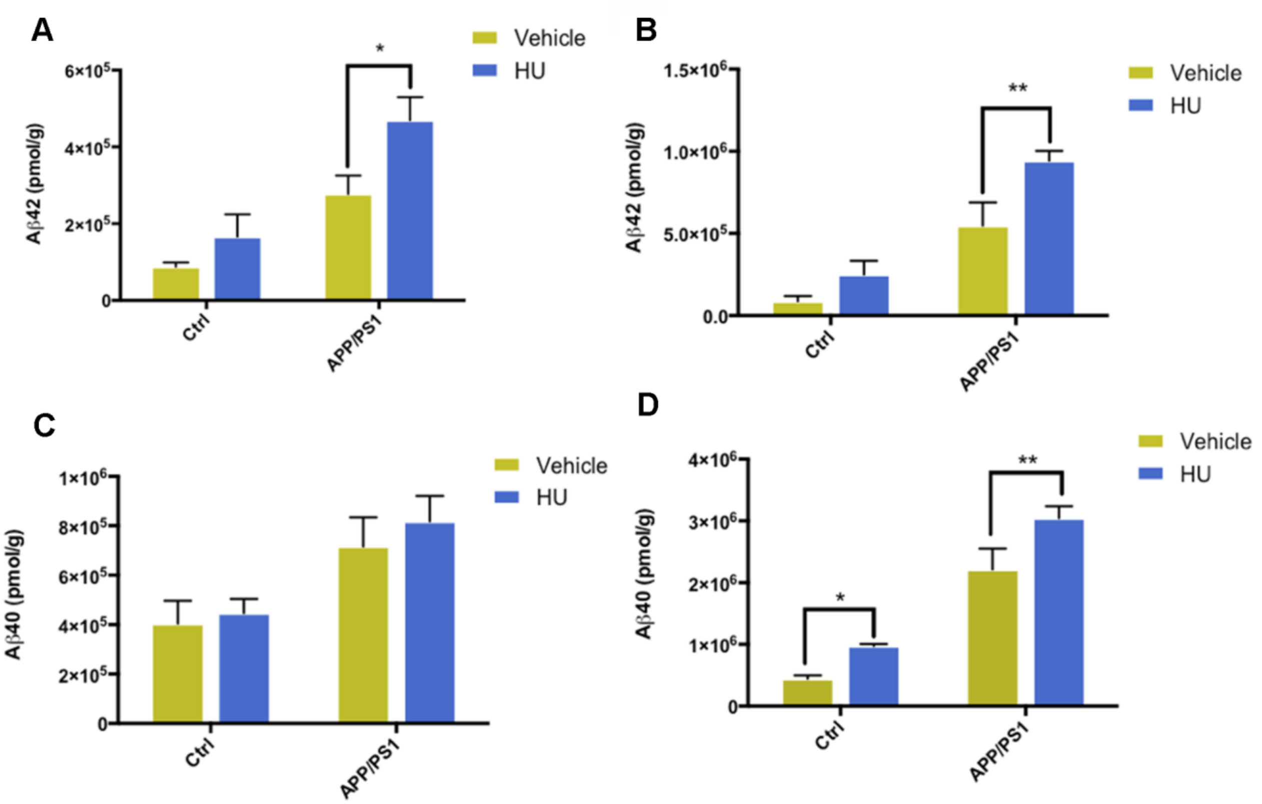

Supplementary Figure 1. Membrane aging increased mRNA levels of Aβ1-40 and Aβ1-42 *in vivo*. The results of ELISA demonstrated that Aβ42 levels increased in the cortex of HU-treated APP/PS1 mice ($P < 0.05$) (**A**). The same trend with more significant change was found in the hippocampus of HU-treated APP/PS1 mice ($P < 0.01$) (**B**). Meanwhile, the levels of Aβ40 hardly changed in the mouse cortex after HU administration (**C**). However, considerably increased levels of Aβ40 were determined after HU treatment in both APP/PS1 and wild-type mice ($P < 0.05$ and $P < 0.01$) (**D**). All the data are expressed as mean ± SD from three independent experiments ($N = 3$). $^*P < 0.05$, $^{**}P < 0.01$. One-way ANOVA was used to determine the statistical significance of the differences.
